# Control-IQ Technology Positively Impacts Patient Reported Outcome Measures and Glycemic Control in Youth with Type 1 Diabetes in a Real-World Setting

**DOI:** 10.1155/2023/5106107

**Published:** 2023-04-12

**Authors:** Caroline Zuijdwijk, Jennilea Courtney, Nicholas Mitsakakis, Lamia Hayawi, Stephanie Sutherland, Dennis Newhook, Alexandra Ahmet, Ellen B. Goldbloom, Karine Khatchadourian, Sarah Lawrence

**Affiliations:** ^1^Division of Endocrinology and Metabolism, Children's Hospital of Eastern Ontario, Ottawa, Ontario, Canada; ^2^CHEO Research Institute, Ottawa, Ontario, Canada; ^3^University of Ottawa, Faculty of Medicine, Ottawa, Ontario, Canada

## Abstract

**Objective:**

To determine the impact of the t:slim X2 insulin pump with Control-IQ technology on the quality of life and glycemic control in youth with type 1 diabetes (T1D) and their parents in a real-world setting. *Research Design and Methods*. We conducted a single-center, prospective study on pediatric patients (6–18 years old) with T1D using a Tandem t:slim X2 pump and initiating Control-IQ technology as part of routine care. Youth (≥8 years) and parents completed validated patient-reported outcome measures (PROMs) at baseline and the end of the study (16 weeks). Glycemic control measures were recorded at baseline and every 4 weeks until the end of the study.

**Results:**

Fifty-nine youth participated; the median (IQR) age was 13.8 (11.1, 15.7) years, and T1D duration was 6.3 (3.1, 8.4) years. INSPIRE scores (evaluating expectations (baseline) and impact (post) of Control-IQ technology) were favorable, unchanged at the end of the study for youth, and lower for parents (*p* = 0.04). Other PROM scores improved by the end of the study with mean (95% CI) differences for youth and parents, respectively, as follows: Diabetes Impact and Device Satisfaction (DIDS) Scale Diabetes Impact −1.08 (−1.51, −0.64) (*p* < 0.001) and −1.41 (−1.96, −0.87) (*p* < 0.001); DIDS Scale Device Satisfaction +0.43 (0.11, 0.74) (*p* = 0.01) and +0.58 (0.31, 0.85) (*p* < 0.001); Hypoglycemia Fear Survey −4.41 (−7.65, −1.17) (*p* = 0.01) and −7.64 (−11.66, −3.62) (*p* < 0.001); and WHO-5 Well-Being Index +5.10 (−1.40, 11.6) (*p* = 0.12) and +9.60 (3.40, 15.8) (*p* = 0.003). The mean time in range increased from 52.6% at baseline to 62.6% (*p* < 0.001) at 4 weeks, sustained to 16 weeks.

**Conclusions:**

Initiation of Control-IQ technology in a real-world setting significantly reduced the impact of diabetes on daily life while simultaneously improving glycemic control. *Trial Registration*. This trial is registered with ClinicalTrials.gov Identifier NCT04838561 (https://www.clinicaltrials.gov/ct2/show/NCT04838561?term=Control-IQ&cond=Type+1+Diabetes&cntry=CA&draw=2&rank=1).

## 1. Introduction

The management of type 1 diabetes (T1D) is rapidly evolving to include technologies aimed at improving metabolic control and decreasing the burden of diabetes management on patients and their families. Automated insulin delivery (AID) systems are at the forefront of these technologies. To date, research evaluating AID systems has primarily been conducted in the context of clinical trials with a focus on improved glycemic control [1–3].

Real-world studies on adults with T1D using the Tandem t:slim X2™ pump with Control-IQ technology, an AID system, have shown improvement in both psychosocial outcomes and persistent achievement of glycemic targets [4, 5]. Pediatric clinical trials assessing the use of AID systems have shown improvement in glycemic control, no increased burden of diabetes management, and improvements in parental sleep, fear of hypoglycemia, and psychosocial measures [6–8]. To our knowledge, no studies have shown a decline in adolescents' self-reported quality of life using this technology, but not all studies have identified improvements [6]. Real-world studies of Control-IQ technology in users aged 6 years or older have shown immediate and sustained (up to 6–12 months) improvements in glycemic control consistent with, or even exceeding, results from randomized controlled trials [9, 10]. In addition to improvement in glycemic control, one real-world pediatric study also demonstrated improvement in the quality of life measures of fear and worry of hypoglycemia in youth and their caregivers [11]. Information is, however, lacking on broader real-world psychosocial outcomes in the pediatric population with the use of Control-IQ technology.

The objective of this study was to determine perceptions of the impact of Control-IQ technology on psychosocial functioning and quality of life in pediatric patients living with T1D and their parents in a real-world setting using patient-reported outcome measures (PROMs) and open-ended exploratory questions. We also assessed the impact of Control-IQ technology on glycemic control.

## 2. Research Design and Methods

We conducted a single-center, prospective study on pediatric patients (6–18 years old) with T1D followed at a tertiary care pediatric hospital in Ottawa, Ontario, Canada. Children and adolescents (hereafter referred to as “youth”) who were already using the Tandem t:slim X2 insulin pump and were initiating Control-IQ technology as part of routine care were invited to participate, along with their parents. Participants were enrolled in the study prior to initiating use of Control-IQ technology, which became available in Canada on March 22, 2021, for individuals already using the Tandem t:slim X2 pump.

Prior to the launch of Control-IQ technology, Basal-IQ technology was available to Tandem t:slim X2 insulin pump users. Both Basal-IQ and Control-IQ technology require the Tandem t:slim X2 pump to be paired with a Dexcom continuous glucose monitor (CGM). Basal-IQ technology uses a predictive low-glucose suspend algorithm, whereas Control-IQ technology is a hybrid closed-loop system that uses an algorithm to automatically adjust insulin delivery in response to predicted high and low glucose levels. Youth were included in this study whether or not they were using Basal-IQ technology prior to the initiation of Control-IQ technology. Youth were excluded if their diabetes duration was less than 1 year to minimize potential impact of the honeymoon period.

The study duration was 16 weeks. At baseline, demographic data and diabetes history were collected from the participants' electronic medical records, and a sociodemographic questionnaire was completed by each participant's parent. Participants received routine care with no additional clinical visits or touchpoints from the diabetes team during the study period. This study received institutional research ethics board approval.

### 2.1. Patient-Reported Outcome Measures

To assess the psychosocial impact of Control-IQ technology, participants completed 4 validated PROMs at baseline and at the end of the study using REDCap (Research Electronic Data Capture), a secure web-based data collection platform. All PROMs were sent to parents and youth aged 8 years or older. For participants 6-7 years of age, only parents completed PROMs. Baseline PROMs were completed before initiation of Control-IQ technology, and at the end of the study, PROMs were completed 16–20 weeks following initiation of Control-IQ technology.

The INsulin dosing Systems: Perceptions, Ideas, Reflections, and Expectations (INSPIRE) questionnaires measure the expectations (baseline) and the impact (post-intervention) of AID systems on users' psychosocial function and quality of life [12]. The youth questionnaires contain 17 items, and the parent questionnaires have 21 items using a 5-point Likert scale from “strongly agree” to “strongly disagree,” with all scores adjusted to be out of 100. The baseline and post-intervention questionnaires include the same questions, with the baseline asking about the *anticipated* impact and the post-intervention about the experienced impact. The questionnaires explore the impact of an AID system on five dimensions: (a) glycemic control, including nocturnal hypoglycemia, (b) activities of daily living, (c) social activities, (d) short- and long-term complications, and (e) overall individual and family quality of life [12, 13]. Higher scores indicate a more favorable anticipated or experienced impact.

The Diabetes Impact and Device Satisfaction (DIDS) Scale uses a 10-point Likert scale to assess (a) the impact of diabetes on the device user's life (4 items) and (b) device-specific satisfaction (7 items) [14]. Scores are expressed as an average (0–10) on each subscale. Parents were directed that “you” means “your child” throughout the questionnaire. On the Diabetes Impact subscale, lower scores are favorable as they indicate less impact from T1D on the individual's life. On the Device Satisfaction subscale, higher scores are favorable as they indicate increased device satisfaction.

The Hypoglycemia Fear Survey (HFS) is a questionnaire that assesses (a) behavior to avoid hypoglycemia (Behavior subscale, 10 items) and (b) worry about hypoglycemia (Worry subscale, 15 items) on a 5-point Likert scale in individuals with T1D [15]. A child version and a parent version were used in the study. The subscales can be scored separately: Behavior (0–40) and Worry (0–60), and an overall score (0–100) is provided. Lower scores (overall and on each subscale) are favorable, indicating less worry about and behaviors to avoid hypoglycemia.

The World Health Organization-Five (WHO-5) Well-Being Index measures current well-being with a score of 0–100 and has been validated for measuring outcomes in clinical trials [16, 17]. Youth responded about their own well-being, whereas we asked parents to respond in relation to their child's well-being. Higher scores indicate greater well-being.

At the end of the study, a parent and/or youth aged 8 years or older were also invited to complete an open-ended questionnaire where they could describe in their own words the impact of Control-IQ technology on sleep, school, activities/sports, mood, and family dynamics. The questionnaires developed by the study team included 8 items for youth and 11 items for parents. Parents were asked about the impact of sleep on both their child and themselves. They were also asked to describe how they felt about diabetes in general, along with any changes they had noticed in their child.

### 2.2. Glycemic Control and Insulin Pump Data

Insulin pumps and Dexcom G6 CGMs were uploaded at baseline and at the end of the study to determine measures of glycemic control (Dexcom Clarity) and insulin pump use (Diasend) at 0, 4, 8, 12, and 16 weeks (14 days of data preceding each time point). Percent time CGM active was used as a proxy for percent time in automation given that this metric was not reported on Diasend, which was the only platform available in Canada for Tandem pump upload during the study period. Data collected at each time point included percent time in target range (TIR) (3.9–10.0 mmol/L or 70–180 mg/dL), percent time below target range (TBR) (<3.9 mmol/L or <70 mg/dL), percent time above target range (TAR) (>10 mmol/L, or >180 mg/dL), average glucose and standard deviation (mmol/L or mg/dL), coefficient of variation (%), glucose management index (GMI) (%), average total daily dose (TDD) of insulin (units), average percent TDD as basal insulin (%), and average number of insulin boluses per day.

### 2.3. Statistical Analysis

Baseline participant characteristics were described using descriptive statistics. We tested if the mean score of post-intervention INSPIRE scores was higher than 60 (determined a priori to indicate a perceived benefit of Control-IQ technology) using the one-sample *t*-test. Pre- and post-mean scores for PROMs were compared using a paired *t*-test. We compared measures of glycemic control and measures of insulin pump management at baseline and every 4 weeks until the end of the study using the paired *t*-test. Mean differences with 95% confidence intervals (CI) were reported. Two-sided *p* values <0.05 were considered statistically significant. All analyses were conducted using R version 4.0.5 [18].

This was a convenience sample with participant numbers determined by the number of patients choosing to initiate Control-IQ technology in the month following its launch in Canada. We anticipated a sample size of 60 participants, sufficient to provide >80% power for detecting a medium-size effect (Cohen's *d* = 0.5).

### 2.4. Qualitative Analysis

Content analysis of the open-ended survey questions was led by 2 members of the research team (DN and SS) with qualitative research expertise. The analysis involved coding each text response [19] and reporting the most frequent codes (response types) for each question. Coding was verified by 2 additional members of the team (SL and CZ). For data display purposes, the top 3 codes (per frequency count) for each survey item are presented in Supplementary Tables, including exemplar quotes where applicable [20].

## 3. Results

Of 79 eligible participants, 63 youth and parent dyads consented to participate. Five participant dyads either did not start or withdrew from the study prior to initiating Control-IQ technology, leaving 59 participant dyads. PROMs were completed by a parent and/or youth in 58 participant dyads at baseline and 54 participant dyads at the end of the study. Baseline characteristics are outlined in Table 1. Fifty-four participants were using Basal-IQ technology on their Tandem t:slim X2 insulin pumps prior to initiation of Control-IQ technology. Our study population was primarily Caucasian with high education and income levels and access to private insurance.

### 3.1. Patient-Reported Outcome Measures

Table 2 details the scores at baseline and the end of the study, as well as mean difference between baseline and end-of-study scores, for each of the 4 validated PROMs. There were no significant differences between the pre- and post-intervention scores on the INSPIRE questionnaire for youth, with a trend toward lower raw scores at the end of the study. For parents, the post-intervention INSPIRE score was lower (*p* = 0.04). The post-intervention INSPIRE mean (SD) scores were 73.0 (11.6) for youth and 70.7 (12.2) for parents, both significantly greater than 60 (*p* < 0.001). Scores for individual items on the INSPIRE questionnaires are shown in Supplementary Tables 1 and 2.

**Figure 1:**
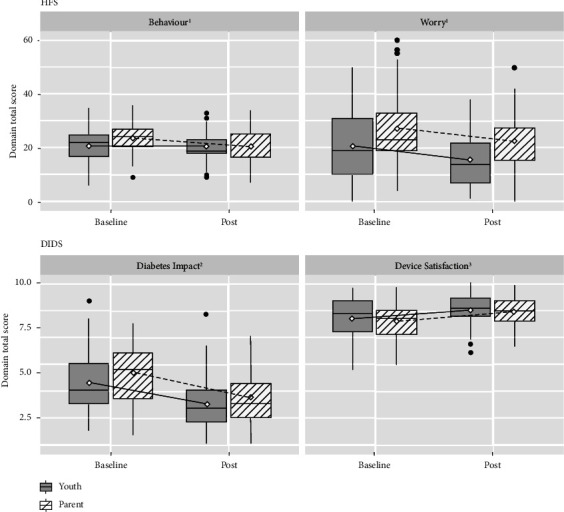
Baseline and 16-week patient-reported outcome measures for parents and youth for the hypoglycemia fear survey (HFS) behavior and worry subscales and diabetes impact and device satisfaction (DIDS) subscales. The diamond shape in each boxplot indicates the mean of the total score of the domain, the horizontal line in the middle of each boxplot indicates the median, and the black line is connecting this mean of each domain at the 2 time points. ^1^Lower score indicates less worry and less behaviors to avoid hypoglycemia. ^2^Lower scores indicate less impact from diabetes on the individual's life. ^3^Higher scores indicate greater satisfaction.

The DIDS Scale identified a statistically significant improvement in both Diabetes Impact and Device Satisfaction subscales at the end of the study in both youth and parents (Table 2 and Figure 1). The mean Diabetes Impact subscale score decreased from 4.4 to 3.2 for youth (*p* < 0.001) and from 5.0 to 3.6 (*p* < 0.001) for parents. The mean Device Satisfaction subscale score increased from 8.0 to 8.5 in youth (*p* = 0.01) and from 7.9 to 8.4 in parents (*p* < 0.001). The most notable changes, in reviewing individual items on the DIDS Scale, were impact on sleep, worry about hypoglycemia and impact on daily activities as reported by both youth and parents (Supplementary Tables 3 and 4).

The mean overall scores of the Hypoglycemia Fear Survey (HFS) decreased from baseline to the end of the study for both youth and parents (Table 2 and Figure 1). For youth, there was a decline on the Worry subscale, but not on the Behavior subscale, consistent with less worry about hypoglycemia on Control-IQ technology, but no changes in their behaviors surrounding this fear. Parents reported an improvement in both Behavior and Worry subscales that were more significant for Behavior. The most notable improvements in the youth HFS were worries around social stigma related to hypoglycemia. For parents, the greatest change was a reduction in behaviors to encourage higher blood glucose levels (Supplementary Tables 5 and 6).

The median scores of the WHO-5 Well-Being Index increased for both youth and parents, but this change was only statistically significant for parent scores. Since parents completed the questionnaire in relation to their child's well-being, results indicate that parents perceived a greater change in their child's well-being than the youth themselves.

### 3.2. Open-Ended Survey Questions

Forty-three participants responded to 1 or more of the open-ended questions at the end of the study (Supplementary Table 7), with the majority commenting on how they were able to achieve better glycemic control with less effort and worry (both parents and youth). The greatest positive impact appeared to be on sleep for both groups. As one parent commented, “…*it gives me more confidence before going to sleep. I can treat early and not have to worry. As a family, it reduced the number of nights we had to wake up to treat a low.*” Other highly cited positive impacts included the benefits of automaticity and nighttime glycemic control. In terms of automaticity, one youth participant expressed, “*I like that you don't have to think about it, it's always on and there is basically a safety net to help*.”

Relatively few participants reported changes for school and sports as the majority of youth noted that they were not at school due to the COVID lockdown. When asked about communication and home dynamics, many youth reported, “*there is less worry and more freedom now*.” The comments provided were positive. When asked what they did not like about Control-IQ technology, most youth indicated that there was nothing to dislike. Eight youth participants commented negatively on the alarms at school, and 4 participants sometimes disagreed with the algorithm and did not like relinquishing control. Hesitations from parents were generally related to adapting to new technology and learning to make adjustments to pump settings and also for exercise. Details are available in Supplementary Table 7.

### 3.3. Glycemic Control and Insulin Pump Data

Percent CGM active (corresponding to percent time in automation) was high overall, with the median (IQR) ranging from 94.0% (87.3, 97.9) to 96.2% (91.9, 98.0) throughout the study.

**Figure 2:**
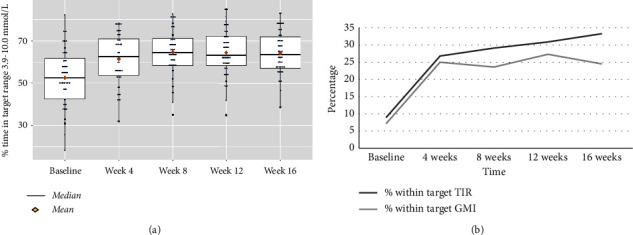
Impact of Control-IQ technology on glycemic outcomes. (a) Percent time in target range (3.9–10.0 mmol/L or 70–180 mg/dL) from baseline to the end of the study. (b) Percentage of participants meeting targets for time in range (TIR ≥70%) and glucose management indicator (GMI <7.0%) from baseline to the end of the study.

Overall, the mean percent TIR improved immediately from 52.6% (baseline) to 62.6% at 4 weeks, with a mean increase (95% CI) of 8.9 (6.5, 11.3), *p* < 0.001, which was sustained to the end of the study (Figure 2(a)). TAR decreased at 4 weeks compared to baseline, with a mean difference (95% CI) of −8.7 (−11.1, −6.3), *p* < 0.001, and was sustained to the end of the study. There was no significant change in TBR at week 4 compared to baseline, with a mean difference (95% CI) of −0.2 (−0.6, 0.3), *p*=0.59. There was also no significant change in the mean glucose, coefficient of variation, or GMI throughout the study. The median GMI (IQR) was 7.7 (7.4, 8.4) at baseline and 7.4 (7.0, 7.7) at the end of the study. The percentage of participants meeting glycemic targets [21] for TIR >70% increased from 8.9% at baseline to a peak of 33.3% of participants at 16 weeks. The percentage of participants with a GMI <7% increased from 7.1% at baseline to a peak of 27.3% at 12 weeks (Figure 2(b)).

The analysis of pump data showed no change in TDD of insulin from baseline to 4 weeks after starting Control-IQ technology. There was, however, a significant increase in the mean (SD) percent of TDD as basal insulin over this same time period from 38.8% (9.5) to 43.3% (10.2), with a mean increase (95% CI) of 4.5% (3.0, 5.6), *p* < 0.001, sustained to 16 weeks. The proportion of insulin given as a bolus declined, but the total number of boluses per day (manual patient-entered and Control-IQ automated correction boluses) increased from baseline to 4 weeks from a mean (SD) of 6.1 (2.3) boluses per day at baseline to 9.1 (3.5) at the end of the study, with a mean difference (95% CI) of 3.2 (2.2, 3.9) boluses per day (*p* < 0.001).

## 4. Discussion

AID systems are designed to improve glycemic control without increasing and, ideally, lessening the burden of T1D management on the patient and family. Herein, we report results of a real-world pediatric study focused primarily on PROMs following initiation of Tandem's Control-IQ technology. Our results show that Control-IQ technology significantly reduced the impact of diabetes on the life of the user while simultaneously improving glycemic control.

The importance of PROMs in evaluating medical devices is increasingly recognized as one way to directly measure the health condition of patients from their own report, without outside interpretation [6, 22]. From a patient perspective, both changes in glycemic control and quality of life are important when considering the use of AID. Cobry et al. reported on PROMs from parents and children in a clinical trial of children with T1D using Control-IQ technology and concluded that those using this technology did not experience increased burden compared with those using an insulin pump and CGM without integration [6]. Our pediatric real-world study and a real-world study of Control-IQ technology in primarily adults [4] report an apparent improvement in device-related satisfaction as well as a reduction in the impact of diabetes on life following the initiation of Control-IQ technology.

The INSPIRE questionnaire is a relatively new validated tool designed to measure user expectations and experience with AID systems. In a clinical trial of Control-IQ technology [6], the INSPIRE score was unchanged from baseline in both parents and children. In the current study, INSPIRE values both before and 16 weeks after initiation of Control-IQ ranged from 70.7 to 76.8 out of 100 points, indicating a positive expectation for and experience with Control-IQ technology. That said, the scores decreased from baseline for both youth and parents, though this change was only significant for parents. In reviewing the individual questions, the magnitude of decline on INSPIRE scores was similar across all dimensions for both youth and parents. The exception was parents reporting a greater gap for the anticipated impact on glycemic control and ease of managing diabetes in social situations. This is despite other measures reporting less fear of hypoglycemia and impact on daily activities and more TIR on CGM reports. Taken together, this suggests that participants liked the technology, but it had less of an impact on their quality of life than anticipated.

Fear of hypoglycemia is a common concern for those living with T1D that has the potential to be impacted by AID technology, particularly at night [23]. Our results show that both youth and parents had a significant decrease in worry about hypoglycemia. Only parents reported a reduction in behaviors aimed at keeping blood glucose levels higher and preventing hypoglycemia. A reduction in fear of hypoglycemia has the potential to increase the likelihood of maintaining euglycemia. This was observed in our study with decreased worry in the context of increased TIR. Other studies have reported a variable change in the HFS with AID. One study found no change in the HFS using a Medtronic AID system [24]. Cobry et al. reported an overall improvement in parental HFS scores with a change in parental behaviors but not in worry with Control-IQ technology and no change in scores for children [6]. In a subsequent clinical trial, this group demonstrated a significant improvement in parental sleep, fear of hypoglycemia, and psychosocial measures with the use of Control-IQ technology [7]. Similarly, a clinical trial demonstrated better well-being and less hypoglycemia fear in caregivers of very young children with T1D on the CamAPS FX hybrid closed-loop system [8]. In a real-world study of youth using Control-IQ technology (91% participants) and the CamAPS FX system (9% participants), Ng et al. found an improvement in HFS worry and behavior scores for both youth and caregivers [11].

Parents in our study reported fewer behaviors to intentionally elevate blood glucose levels and less worry about hypoglycemia overnight with improved sleep. It is interesting to note, however, that 91.5% of participants in our study were already using Basal-IQ technology at baseline. We were therefore surprised that parents reported fewer behaviors to intentionally elevate blood glucose levels and less worry about hypoglycemia overnight when their children transitioned from Basal-IQ to Control-IQ technology, as both systems lower insulin delivery in response to predicted hypoglycemia. This finding may be related to education provided to parents (through online upgrade modules) about Control-IQ technology prior to initiation, providing a greater awareness of the safety of the AID system. An even greater decline in fear of hypoglycemia might have been expected if patients had not had access to Basal-IQ technology prior to initiation of Control-IQ technology.

Qualitative studies have explored what is important to youth and parents in an AID system [25, 26]. Naranjo et al. reported on what end users want from AID systems. In that study, children and adolescents identified factors specific to social contexts, while parents were more concerned about accuracy and glycemic control [25]. This is reflected in our findings, in that much of the benefit reported by youth on the HFS was in reducing concerns around social stigma and missing activities due to diabetes.

The improvement in glycemic outcomes in our real-world study was comparable to that observed in clinical trials and real-world studies of Control-IQ technology [3, 9, 10, 27, 28], with a mean increase of 8.9% for TIR (from 52.6% to 62.6%), with no significant change in frequency of hypoglycemia. Glycemic control improved immediately in the first 4 weeks and was sustained for the remaining 12 weeks of observation. TBR did not change, which was likely secondary to preexisting use of Basal-IQ technology for most participants. This is consistent with findings of Marigliano et al., who found no change in TBR in patients transitioning from Basal-IQ to Control-IQ technology [29]. While the TDD of insulin was not affected, the overall percent of TDD insulin as basal increased by 4.5%, and there were significantly more boluses, likely related to the automated correction boluses delivered through the Control-IQ algorithm.

Limitations of this study include its relatively small size, short duration of follow-up, and a risk of selection bias. The province of Ontario has publicly funded health care and insulin pump funding that theoretically supports equitable access. However, CGM supplies were not publicly funded at the time of this study, and 90% of participants had private health insurance to support costs. Participants were generally from well-educated, middle- to high-income households. Taken together, this may limit the generalizability of these results. However, previous studies have shown that the benefit of AID systems may be equal or greater in populations with more limited resources and higher GMI at baseline [30]. In this study, improvements in glycemia were demonstrated in a high-resource population, with a baseline GMI of 7.7%, suggesting that there may be even greater benefits observed in a more diverse population. Similarly, our study looked at the outcomes of the initiation of Control-IQ technology in a patient population where 91.5% were already using Basal-IQ technology. It is possible that even greater psychosocial and glycemic benefits may be observed in patients transitioning to Control-IQ technology from other less-intensive insulin regimens (i.e., multiple daily injections ± CGM or stand-alone insulin pumps).

Finally, we acknowledge certain limitations due to data availability during the study period. Percent time CGM active was used as a proxy for percent time of Control-IQ use (given that this metric was not directly available on uploads), which are not always the same. However, percent time CGM active was high and suggests that Control-IQ technology was being used. Additionally, due to limited access to HbA1c measurements during the COVID-19 pandemic, we relied on GMI and TIR. While these measures are increasingly recognized as outcomes for glycemic control [31], GMI and HbA1c are not completely interchangeable [32].

## 5. Conclusions

Initiation of the Control-IQ technology AID system in a real-world setting significantly improved measures of psychosocial functioning and quality of life in youth with T1D and their parents while also improving glycemic control. The impact of Control-IQ technology on quality of life, as measured by the INSPIRE questionnaire, was high, though lower parent scores at the end of the study indicate the importance of managing expectations. Overall, satisfaction with Control-IQ technology was high, and patients reported improved glycemic control with less fear of hypoglycemia and a reduction in the burden of diabetes on their lives. Larger real-world studies with diverse patient populations are warranted to further assess the generalizability of these findings.

## Figures and Tables

**Table 1:** Baseline participant characteristics.

Variables	*N*	Missing (%)	Value
Age (years), median (IQR)	59	0 (0.0)	13.8 (11.1, 15.7)
Sex (male), number (%)	59	0 (0.0)	28 (47.5)
Duration of T1D (years), median (IQR)	59	0 (0.0)	6.3 (3.1, 8.4)
Duration of insulin pump use (years), median (IQR)	59	0 (0.0)	1.97 (0.8, 5.8)
Current uses of the continuous glucose monitor (yes), frequency (%)	59	0 (0.0)	58 (98.3)
Current use of Basal-IQ technology (yes), frequency (%)	59	0 (0.0)	54 (91.5)
Ethnicity (self-identified), frequency (%)	51	8 (13.6)	
White			42 (82.3)
Other (included Arab, black, Japanese, South Asian, preferred not to answer)			9 (17.7)
With whom does the child/youth live majority of the time? frequency (%)	51	8 (13.6)	
Living with both parents			45 (88.2)
Other family structure			6 (11.8)
Highest level of education, parents	51	8 (13.6)	
High school certificate or equivalent			1 (2.0)
Apprenticeship certificate or equivalent			1 (2.0)
College or other nonuniversity certificate/diploma			13 (25.5)
University certificate or diploma			18 (35.3)
Postgraduate degree			18 (35.3)
Total household income (combined), frequency (%)	48	11 (18.6)	
Less than $50.000			3 (6.3)
$50,000 to $99,999			9 (18.7)
More than $100,000			36 (75.0)
Youth has private health insurance (yes), frequency (%)	52	7 (11.9)	48 (92.3)

**Table 2:** Patient-reported outcome measures (PROM) at baseline and 16 weeks after initiation of Control-IQ technology.

PROM (total score)	Youth						Parents					
	Pre		Post		Mean difference (post-baseline) (95% CI)	*p* value^1^	Pre		Post		Mean difference (postbaseline) (95% CI)	*p* value^1^
	*N*	Mean (SD) or median (IQR)	*N*	Mean (SD) or median (IQR)			*N*	Mean (SD) or median (IQR)	*N*	Mean (SD) or median (IQR)		
INSPIRE (1–100)	49	76.8 (14.0)	49	73.0 (11.6)	−3.98 (−8.51, 0.54)	0.08	54	74.6 (9.2)	45	70.7 (12.2)	−3.49 (−6.88, −0.10)	0.04
DIDS												
Diabetes Impact (0–10)	49	4.4 (1.9)	49	3.2 (1.4)	−1.08 (−1.51, −0.64)	<0.001	53	5.0 (1.7)	43	3.6 (1.4)	−1.41 (−1.96, −0.87)	<0.001
Device Satisfaction (0–10)	49	8.0 (1.2)	49	8.5 (1.0)	0.43 (0.11, 0.74)	0.01	53	7.9 (0.9)	43	8.4 (0.8)	0.58 (0.31, 0.85)	<0.001
HFS												
Overall	49	40.3 (15.4)	49	36.2 (13.0)	−4.41 (−7.65, −1.17)	0.01	52	51.3 (16.2)	43	42.9 (15.4)	−7.64 (−11.66, −3.62)	<0.001
Behavior (0–40)	49	20.4 (6.5)	49	20.5 (5.3)	−0.46 (−1.95, 1.04)	0.54	52	23.9 (5.2)	43	20.5 (6.3)	−2.87 (−4.37, −1.38)	<0.001
Worry (0–60)	49	19.9 (13.1)	49	15.7 (10.6)	−3.96 (−6.51, −1.40)	0.003	52	27.4 (13.0)	43	22.4 (11.1)	−4.77 (−8.09, −1.45)	0.01
WHO-5 (0–100)	49	60.0 (48.0, 76.0)^2^	49	72.0 (60.0, 80.0)^2^	5.1 (−1.4, 11.6)	0.12	56	68.0 (55.0, 76.0)^2^	43	76.0 (68.0, 80.0)^2^	9.6 (3.4, 15.8)	0.003

^1^ *t*-test for paired samples to compare the difference in means. ^2^Median (interquartile range) is reported.

## Data Availability

The datasets generated during and analyzed in the current study are available from the corresponding author upon reasonable request.

## Additional Points

*Article Highlights*. (i) This study adds to a small growing body of the literature focused on real-world patient-reported outcome measures and glycemic control with the use of automated insulin delivery systems in the pediatric population. (ii) Initiation of Control-IQ technology in a real-world setting significantly improved fear of hypoglycemia and impact of diabetes on daily life in youth with type 1 diabetes and their parents. (iii) Results of the INSPIRE questionnaire show a positive impact of Control-IQ technology on quality of life in both youth and their parents and indicate the importance of managing expectations. (iv) Glycemic control improved immediately at 4 weeks post-initiation of Control-IQ technology and was sustained to the end of the study (16 weeks) in a real-world setting.

## Supplementary Materials

Supplementary Materials Table 1: Mean total and item scores for the INSPIRE youth questionnaire at baseline and post-intervention. Table 2: Mean total and item scores for the INSPIRE parent questionnaire at baseline and post-intervention. Table 3: Mean scores for DIDS items for youth (baseline and the end of the study). Table 4: Mean scores for DIDS items for parents (baseline and the end of the study). Table 5: Mean scores for HFS Behavior and Worry subscales for youth (baseline and the end of the study). Table 6: Mean scores for HFS Behavior and Worry subscales for parents (baseline and end of study). Table 7: Qualitative responses to open-ended questions.
