# Supplementary material for: Control-IQ Technology Positively Impacts Patient Reported Outcome Measures and Glycemic Control in Youth with Type 1 Diabetes in a Real-World Setting
Source: Pediatr Diabetes. 2023 Apr 12;2023:5106107. doi: 10.1155/2023/5106107 (PMC12020727; doi:10.1155/2023/5106107)
Supplement: Supplementary Materials — Table 1: Mean total and item scores for the INSPIRE youth questionnaire at baseline and post-intervention. Table 2: Mean total and item scores for the INSPIRE parent questionnaire at baseline and post-intervention. Table 3: Mean scores for DIDS items for youth (baseline and the end of the study). Table 4: Mean scores for DIDS items for parents (baseline and the end of the study). Table 5: Mean scores for HFS Behavior and Worry subscales for youth (baseline and the end of the study). Table 6: Mean scores for HFS Behavior and Worry subscales for parents (baseline and end of study). Table 7: Qualitative responses to open-ended questions. [file 5106107.f1.docx]

**SUPPLEMENTARY TABLES:**

### Table 1: Mean total and item scores for INSPIRE Youth questionnaire at baseline and post-intervention

| **Items** | **N Baseline** | **Baseline**  **Mean (SD)** | **N post** | **Post**  **Mean (SD)** |
| --- | --- | --- | --- | --- |
| INSPIRE total score | 49 | 76.77 (14.01) | 49 | 73.02 (11.55) |
| More hopeful about my future with use of AID dosing | 49 | 3.27 (0.64) | 49 | 3.12 (0.73) |
| Worry less about diabetes with AID. | 49 | 2.90 (0.92) | 49 | 2.98 (0.80) |
| Reduce my family’s concerns about my diabetes | 49 | 3.02 (0.78) | 49 | 2.96 (0.98) |
| Easier for me to do the things I want to do without diabetes getting in the way | 49 | 3.10 (0.77) | 49 | 2.78 (0.87) |
| Decrease how often I have low glucose levels | 49 | 2.78 (0.87) | 49 | 2.90 (0.92) |
| Decrease how often I have high glucose levels | 49 | 3.24 (0.69) | 49 | 2.94 (0.83) |
| Help me stay in my target glucose range more often | 49 | 3.24 (0.78) | 49 | 3.29 (0.68) |
| Improve my A1c to target level | 49 | 3.24 (0.75) | 47 | 2.89 (0.79) |
| Easy to eat when I want | 49 | 2.73 (1.06) | 49 | 2.67 (0.94) |
| Easy to exercise when I want | 49 | 2.71 (0.94) | 49 | 2.61 (0.89) |
| Managing diabetes easy when I am at school or work | 49 | 3.35 (0.75) | 49 | 3.16 (0.72) |
| Managing diabetes easy when I am with my friends | 49 | 3.16 (0.92) | 49 | 3.02 (0.69) |
| Help me manage sick days. | 49 | 3.02 (0.78) | 49 | 2.39 (0.61) |
| Help me sleep better | 49 | 3.02 (1.07) | 46 | 2.78 (0.96) |
| Fewer lows during the night with AID | 48 | 3.04 (0.80) | 49 | 2.92 (0.91) |
| Improve my overall quality of life | 49 | 3.20 (0.74) | 49 | 3.12 (0.67) |
| Improve my family’s overall quality of life | 49 | 3.14 (0.84) | 49 | 3.10 (0.74) |

### Table 2: Mean total and item scores for INSPIRE Parent questionnaire at baseline and post-intervention

| **Items** | **Number**  **Baseline** | **Baseline**  **Mean (SD)** | **Number Post** | **Post**  **Mean (SD)** |
| --- | --- | --- | --- | --- |
| Total INSPIRE score | 54 | 74.59 (9.19) | 45 | 70.67 (12.24) |
| Hopeful about my child’s future with use of (AID) | 53 | 3.51 (0.61) | 45 | 3.22 (0.90) |
| Worry less about diabetes with AID | 54 | 3.06 (0.86) | 45 | 3.20 (0.76) |
| Reduce my family’s concerns about my child’s diabetes | 53 | 3.00 (0.78) | 45 | 2.98 (0.97) |
| Easier for my child to do what they want to do without diabetes getting in the way | 54 | 3.19 (0.73) | 44 | 2.98 (0.73) |
| Decrease how often my child has low glucose levels | 53 | 2.70 (0.89) | 45 | 3.00 (0.77) |
| Decrease how often my child has high glucose levels | 54 | 3.26 (0.48) | 44 | 2.84 (0.91) |
| Help my child stay in his/her target range more often | 54 | 3.33 (0.48) | 45 | 3.07 (0.81) |
| Improve my child’s A1c to target level | 54 | 3.37 (0.52) | 44 | 2.82 (0.97) |
| Easy to eat when my child wants | 54 | 2.44 (1.02) | 45 | 2.53 (0.99) |
| Easy to exercise when my child wants | 54 | 2.59 (0.81) | 45 | 2.42 (0.81) |
| Managing diabetes easy when my child is at school or work | 54 | 3.19 (0.52) | 45 | 2.98 (0.84) |
| Managing diabetes easy when it comes to my child’s social life/being with friends | 54 | 3.06 (0.66) | 45 | 2.82 (0.81) |
| Help me manage my child’s sick days | 54 | 2.80 (0.63) | 45 | 2.42 (0.72) |
| Help me sleep better | 54 | 3.19 (0.70) | 45 | 3.16 (0.95) |
| My child will have fewer lows during the night with AID | 54 | 2.83 (0.75) | 45 | 3.20 (0.87) |
| Improve my overall quality of life | 54 | 3.13 (0.65) | 45 | 3.09 (0.76) |
| Improve my family’s overall quality of life | 53 | 3.19 (0.62) | 45 | 3.00 (0.67) |
| Managing diabetes easy when my child is driving (for those who drive) or when traveling | 54 | 2.85 (0.63) | 43 | 2.51 (0.74) |
| Help my child manage diabetes if she/he chooses to drink alcohol | 53 | 2.25 (0.78) | 43 | 2.05 (0.65) |
| Help my child manage diabetes if pregnant | 52 | 2.40 (0.75) | 41 | 2.07 (0.57) |
| Reduce my child’s risk of long-term complications | 54 | 3.30 (0.54) | 45 | 2.91 (0.70) |

### Table 3: Mean scores for DIDS items for Youth (baseline and end of study)

| **Item** | **Number Baseline** | **Baseline**  **Mean (SD)** | **Number**  **Post** | **Post**  **Mean (SD)** |
| --- | --- | --- | --- | --- |
| **Device Satisfaction (items 1-7) [Higher scores mean higher satisfaction]** |  |  |  |  |
| How satisfied are you with your current insulin delivery device? | 49 | 7.94 (1.28) | 49 | 8.47 (1.37) |
| How much do you trust your current insulin delivery device? | 49 | 7.98 (1.49) | 49 | 8.59 (1.04) |
| Is easy to use? | 49 | 8.67 (1.71) | 49 | 8.96 (0.98) |
| Helps me have good blood glucose control | 49 | 7.71 (1.65) | 49 | 8.22 (1.50) |
| Is a hassle to use | 49 | 7.47 (2.60) | 48 | 8.08 (2.15) |
| Helps me feel more in control of my diabetes | 49 | 7.69 (1.81) | 49 | 7.96 (1.73) |
| Is too complicated | 49 | 8.63 (2.18) | 49 | 9.06 (1.36) |
| **Diabetes Impact (items 8-11), [Lower scores mean less impact on life]** |  |  |  |  |
| Have a bad night sleep due to diabetes? | 49 | 4.43 (2.29) | 49 | 3.31 (1.71) |
| Wake up at night to treat a low blood glucose? | 49 | 4.24 (2.39) | 49 | 3.51 (2.04) |
| Worry about going low? | 49 | 4.73 (2.47) | 49 | 3.47 (2.18) |
| Miss work, school, chores, or other responsibilities due to diabetes? | 49 | 4.12 (2.47) | 49 | 2.65 (2.04) |

### Table 4: Mean scores for DIDS items for Parents (baseline and end of study)

| **Item** | **Number Baseline** | **Baseline**  **Mean (SD)** | **Number Post** | **Post**  **Mean (SD)** |
| --- | --- | --- | --- | --- |
| **Device Satisfaction (items 1-7) [Higher values mean higher satisfaction]** |  |  |  |  |
| How satisfied are you with your current insulin delivery device? | 53 | 7.83 (1.19) | 43 | 8.44 (0.98) |
| How much do you trust your current insulin delivery device? | 53 | 8.00 (1.07) | 43 | 8.49 (0.88) |
| Is easy to use. | 53 | 8.21 (1.21) | 43 | 8.33 (1.43) |
| Helps me have good blood glucose control | 53 | 7.42 (1.35) | 43 | 8.16 (1.23) |
| Is a hassle to use | 53 | 7.49 (2.18) | 43 | 8.53 (1.30) |
| Helps me feel more in control of my diabetes | 53 | 7.62 (1.38) | 43 | 7.88 (1.38) |
| Is too complicated | 53 | 8.42 (1.66) | 44 | 8.61 (1.50) |
| **Diabetes Impact (items 8-11), [Lower scores mean less impact on life]** |  |  |  |  |
| Have a bad night sleep due to diabetes? | 53 | 5.91 (2.37) | 43 | 3.67 (1.67) |
| Wake up at night to treat a low blood glucose? | 53 | 4.83 (1.87) | 43 | 3.74 (1.56) |
| Worry about going low? | 53 | 5.64 (2.65) | 43 | 4.47 (2.10) |
| Miss work, school, chores, or other responsibilities due to diabetes? | 52 | 3.44 (2.26) | 43 | 2.58 (1.61) |

### Table 5: Mean scores for HFS Behavior and Worry subscales for Youth (baseline and end of study)

| **Items** | **Number Baseline** | **Baseline**  **Mean (SD)** | **Number Post** | **Post**  **Mean (SD)** |
| --- | --- | --- | --- | --- |
| **HFS-Behavior Total Score (0-40)** |  |  |  |  |
| Eat large snacks at bedtime | 49 | 1.59 (0.93) | 49 | 1.71 (0.98) |
| Try not to be by myself when my sugar is likely to be low | 49 | 1.63 (1.29) | 49 | 1.82 (1.20) |
| Keep blood sugars to be a little high to be on the safe side | 49 | 1.61 (1.08) | 49 | 1.61 (0.84) |
| Keep my sugar higher when I will be alone for a while | 49 | 1.22 (1.23) | 49 | 1.06 (1.09) |
| Eat something as soon as I feel the first sign of low blood sugar | 49 | 2.39 (1.15) | 49 | 2.49 (1.26) |
| Take less insulin when I think my sugar might get too low | 49 | 1.86 (1.21) | 49 | 1.69 (1.14) |
| Keep my blood sugar higher when I am going to be away from my parents | 49 | 1.35 (1.18) | 48 | 1.19 (1.02) |
| Carry some kind of sugar, drink or food with me | 49 | 3.49 (0.79) | 49 | 3.65 (0.60) |
| Try not to do exercise when I think my sugar is low | 49 | 2.67 (1.31) | 49 | 2.65 (1.11) |
| Check my sugar often when I am away from home | 49 | 2.63 (1.11) | 49 | 2.63 (1.13) |
| **HFS-Worry Total Score (0-60)** |  |  |  |  |
| Not recognizing that my blood sugar is low | 49 | 1.24 (1.23) | 49 | 0.98 (0.99) |
| Not having food, fruit or juice with me when my blood sugar gets low | 49 | 1.80 (1.17) | 49 | 1.41 (0.93) |
| Feeling dizzy or passing out in public because of low blood sugar | 49 | 1.55 (1.34) | 49 | 1.00 (0.98) |
| Having a reaction while asleep | 49 | 1.31 (1.19) | 49 | 1.22 (1.14) |
| Embarrassing myself because of low blood sugar | 49 | 1.24 (1.33) | 49 | 0.92 (1.10) |
| Having a reaction while I am by myself | 49 | 1.39 (1.20) | 49 | 1.27 (1.08) |
| Appearing to be stupid or clumsy in front of other people | 49 | 1.35 (1.45) | 49 | 0.90 (1.01) |
| Losing control because of low blood sugar | 49 | 1.29 (1.17) | 49 | 0.78 (0.77) |
| No one being around to help me during a reaction | 49 | 1.47 (1.31) | 49 | 1.16 (1.11) |
| Making a mistake or having an accident at school because of al ow sugar | 48 | 1.50 (1.41) | 49 | 1.14 (1.04) |
| Getting in trouble at school because of something that happens when my sugar is low | 49 | 1.10 (1.40) | 48 | 1.10 (1.19) |
| Having seizures | 49 | 0.73 (1.08) | 49 | 0.59 (0.81) |
| Getting long-term complications from frequent low blood sugars | 49 | 1.04 (1.17) | 49 | 0.80 (0.91) |
| Feeling dizzy or woozy when my sugar is low | 49 | 1.55 (1.21) | 49 | 1.22 (0.94) |
| Having a reaction | 49 | 1.37 (1.24) | 49 | 1.22 (1.03) |

### Table 6: Mean scores for HFS Behavior and Worry subscales for Parents (baseline and end of study)

| **Items** | **Number Baseline** | **Baseline**  **Mean (SD)** | **Number Post** | **Post**  **Mean (SD)** |
| --- | --- | --- | --- | --- |
| **HFS-Behavior Total Score (0-40)** |  |  |  |  |
| Have my child eat large snacks at bedtime | 52 | 1.35 (1.06) | 42 | 1.33 (0.93) |
| Avoid having my child be alone when his/her sugar is likely to be low | 52 | 2.75 (1.14) | 42 | 2.29 (1.22) |
| Allow my child’s blood sugars to be a little high to be on the safe side | 52 | 1.98 (0.73) | 43 | 1.56 (0.91) |
| Keep my child’s sugar higher when he/she will be alone for a while | 52 | 1.73 (1.14) | 43 | 1.37 (1.07) |
| Have my child eat something as soon as he/she feels the first sign of low blood sugar | 52 | 2.63 (1.14) | 43 | 2.72 (1.01) |
| Reduce my child’s insulin when I think his/her sugar might get too low | 52 | 2.33 (0.98) | 43 | 1.42 (1.22) |
| Keep my child’s blood sugar higher when he/she plans to be away from me for a while | 52 | 1.79 (1.13) | 42 | 1.45 (1.06) |
| Have my child carry fast-acting sugar | 52 | 3.79 (0.46) | 43 | 3.84 (0.43) |
| Have my child avoid a lot of exercise when I think his/her sugar is low | 51 | 2.43 (1.08) | 43 | 2.09 (1.19) |
| Check my child’s sugar often when he/she is away from home | 52 | 3.13 (0.79) | 43 | 2.56 (1.22) |
| **HFS-Worry Total Score (0-60)** |  |  |  |  |
| My child not recognizing/realizing that he/she is having a low blood sugar | 52 | 2.19 (1.16) | 43 | 2.02 (1.28) |
| My child not having food, fruit or juice with him/her | 52 | 2.25 (1.10) | 43 | 2.14 (1.17) |
| My child feeling dizzy or passing out in public because of low blood sugar | 51 | 1.94 (1.33) | 43 | 1.56 (1.14) |
| My child having a reaction while asleep | 52 | 2.52 (1.16) | 43 | 2.14 (1.13) |
| My child embarrassing self or friends/family because of low blood sugar | 52 | 0.83 (1.04) | 43 | 0.42 (0.73) |
| My child having a reaction while alone | 52 | 2.42 (1.07) | 43 | 2.05 (1.11) |
| My child appearing to be stupid or clumsy in front of other people | 52 | 0.73 (1.12) | 43 | 0.30 (0.60) |
| My child losing control because of low blood sugar | 51 | 1.45 (1.27) | 43 | 1.21 (1.06) |
| No one being around to help my child during a reaction | 51 | 2.39 (1.11) | 43 | 2.23 (1.27) |
| My child making a mistake or having an accident at school | 52 | 1.37 (1.30) | 43 | 1.12 (1.07) |
| My child getting a bad evaluation at school because of something that happens when his/her sugar is low | 52 | 1.60 (1.33) | 43 | 1.30 (1.21) |
| My child having seizures or convulsions | 52 | 1.81 (1.41) | 42 | 1.14 (1.28) |
| My child getting long-term complications from frequent low blood sugars | 51 | 2.00 (1.17) | 43 | 1.53 (0.96) |
| My child feeling lightheaded or faint | 52 | 2.04 (1.10) | 43 | 1.63 (0.98) |
| My child having a reaction | 52 | 2.06 (1.18) | 43 | 1.65 (0.97) |

**Table 7: Qualitative Responses to Open Ended Questions**

**Section 1. Youth Response Data**

| **Question 1: How has using Control-IQ affected you?** | |
| --- | --- |
| **Response codes and exemplar quotes** (n = 43) | **Frequency counts*** |
| **Improved control**  *Control IQ has offered me so much control especially when it comes to activities and work. The prevention measure it takes allows me to continue my workout or activities or reassures me if I cannot treat right away. It has done miracles for my post low treatment highs and it has prevented sudden post low blood sugar rises. The sleep mode is a bonus as well. It allows me to worry less about low blood sugar during the evenings* | 22 |
| **Less worry**  *It has affected me in a positive way. It made me worry less about managing my diabetes and focus more on what I want to do in my daily life* | 16 |
| **Improved ease of management**  *Control-IQ has made managing my blood glucose levels easier for me* | 9 |

********Number of times mentioned in text; in some instances, frequency may exceed 100% as a single response could receive more than one code.*

| **Question 2: What do you like about Control-IQ?** | |
| --- | --- |
| **Response codes and exemplar quotes** (n = 43) | **Frequency counts** |
| **Ease of management**  *I like that I don't really have to do much for it and it is helpful to have. It is helpful for keeping my blood sugar in range* | 22 |
| **Safe**  *I love that extra feeling of safety. I know that if I'm off on my carbs a little, it will help me. I know that if I'm dropping it will try it's best to stop it. It just removes that little extra fear that used to always be present* | 7 |
| **Less worry**  *I like the idea of being able to worry less about high and low blood sugar and being able to rely more on my pump instead of needing to take it out in public* | 3 |

| **Question 3: What don't you like about Control IQ?** | |
| --- | --- |
| **Response codes and exemplar quotes** (n = 40) | **Frequency counts** |
| **Nothing (like everything)**  *There is nothing I don’t like about Control IQ* | 17 |
| **Alarms / beeping**  *I don't like how it had its own Alarm. I find it hard to deal with at school and work when it keeps going off* | 8 |
| **Lack of control**  *Sometimes I disagree with the way it proceeds with my treatment. It's not because I don't trust it but mainly because I want all the control. It's still hard to let go of the rains* | 4 |

| **Question 4: How has Control-IQ changed your sleep?** | |
| --- | --- |
| **Response codes and exemplar quotes** (n = 46) | **Frequency counts** |
| **Less waking up in the night / more sleep**  *I get better sleep because Control IQ helps me have less lows or highs so my mom and dad don't have to wake me up as much in the night* | 25 |
| **Less lows**  *Gives me more confidence before going to sleep. I can treat early and not have to worry. As a family, it reduced the number of nights we had to wake up to treat a low* | 7 |
| **Little to no change in sleep**  *It has not changed my sleep very much* | 7 |

| **Question 5: How has it changed things for you with school?** | |
| --- | --- |
| **Response codes and exemplar quotes** (n = 42) | **Frequency counts** |
| **COVID lockdown (respondent not physically in school)**  *I didn’t go to school this year because to covid. I did do online school* | 16 |
| **Less worry / better focus on school**  *I'm way more focused at school. I also don't have to sit out on the sides as often because it does such a great job keeping me in range* | 9 |
| **No change**  *It hasn’t really changed* *anything* | 7 |

| **Question 6: How has it changed things for you during activities? (day to day activity and/or organized sports)** | |
| --- | --- |
| **Response codes and exemplar quotes** (n = 42) | **Frequency counts** |
| **Less lows**  *It has changed things for me so that when I am doing my favourite sports I don't go low as much as I used to and I don't need as much sugar when I do* | 11 |
| **More exercise / activity**  *Control-IQ has changed things during my day-to-day activities such as sports because again it is trying to prevent my highs or lows while I'm out on the ice or running or doing really anything physical* | 11 |
| **Little change**  *It hasn’t changed much* | 9 |

| **Question 7: How has Control-IQ changed how you feel about diabetes?** | |
| --- | --- |
| **Response codes and exemplar quotes** (n = 32) | **Frequency counts** |
| **Ease of management**  *It has not changed how I feel about diabetes but has made it easier to manage* | 8 |
| **No change**  *It has not changed how I feel about diabetes* | 6 |
| **More confidence/optimism**  *It makes me a bit more confident in how I see things and how the future will come out with more stuff to help* | 5 |

| **Question 8: Has Control-IQ changed how much fun you have together as a family? How you get along with your parents?**  **Or the amount of time you spend talking about diabetes? Please tell us more about this.** | |
| --- | --- |
| **Response codes and exemplar quotes** (n = 38) | **Frequency counts** |
| **Communication**  *It has allowed me to talk to my friends more about diabetes awareness* | 6 |
| **Less worry**  *I'm arguing less with my mom and what my blood sugar is because I'm having less lows. Overall things are happier in my house and a lot of stress has been relived. Especially with my mom and her worrying* | 5 |
| **More freedom**  *It has helped my parents give me some more space with my diabetes* | 4 |

**Section 2. Parent Response Data**

| **Question 1: How has your child's use of Control-IQ affected you?** | |
| --- | --- |
| **Response codes and exemplar quotes** (n = 39) | **Frequency counts** |
| **Improved control**  *Much easier to control diabetes with Control-IQ*  *We find our son has fewer lows and we use temp vassals less frequently* | 15 |
| **Less worry**  *Yes, it did affect me in a good way, I am feeling less worry about her diabetes control.* | 12 |
| **Improved sleep**  *I see less hypo’s overnight and improved sleep cycles for all the family.* | 10 |

| **Question 2: What do you like about Control-IQ?** | |
| --- | --- |
| **Response codes and exemplar quotes** (n = 40) | **Frequency counts** |
| **Improved control (includes automaticity and nighttime control)**  *I like that you don’t have to think about it, it’s always on and there and is basically a safety net to help*  *Automatically giving insulin to treat oncoming highs*  *Keeping my son more time in range*  *That it works automatically!* | 31 |
| **Easy to use** | 2 |

| **Question 3: What don’t you like about Control-IQ** | |
| --- | --- |
| **Response codes and exemplar quotes*** (n = 33) | **Frequency counts** |
| **Adapting to new technology**  *It is difficult to manage when you give him a pen needle to correct as the pump does not factor that insulin in iob unless you disconnect tube and waste the equivalent dose[….] Something to get used to.*  *It's a little harder to know how to adjust her doses but that might just be because I have a hard time understanding all the information I see on diasend.*  *Having to check for a correction bolus when blood sugars are high and we are unable to bolus.* | 12 |
| **Difficulty adjusting settings**  *It's tricky to get the calibration right. The automatic overtreatment of potential highs can sometimes result in lows.*  *I don't like that it still uses manually entered basal rates and correction ratios... instead of adapting or even giving suggestions. I sometimes think the algorithm could be more aggressive… there have been several nights where my son has been high and it has taken the entire night for the pump to bring him back down to range.* | 8 |
| **Loss of connection with Dexcom** | 1 |
| **Alarms** | 1 |

***Note:** many responses reported no dislikes or were not applicable (n=12)

| **Question 4: How has Control-IQ changed your own sleep?** | |
| --- | --- |
| **Response codes and exemplar quotes** (n = 39) | **Frequency counts** |
| **Better sleep (includes more sleep and less interrupted sleep)**  *Sleeping better and longer*  *GAME CHANGER! I don’t wake up to check his levels!!!*  *Helped tremendously. Most nights there is no need to treat highs or lows.* | 29 |
| **No changes in sleep** | 5 |

| **Question 5: How has Control-IQ affected your child/teen’s sleep?** | |
| --- | --- |
| **Response codes and exemplar quotes** (n = 39) | **Frequency counts** |
| **Better sleep (includes less interruptions/disturbances during night)**  *He is sleeping much better I think.*  *She gets more sleep because we don’t have to wake her up as often.* | 21 |
| **No change in sleep** | 7 |
| **Better mood in morning**  *He’s waking up in a much better mood due to having better readings at night.* | 4 |

| **Question 6: How has it changed things with school?** | |
| --- | --- |
| **Response codes and exemplar quotes** (n = 38) | **Frequency counts** |
| **No change noticed** (also includes responses where child is not physically at school so no opportunity to observe change) | 26 |
| **School easier because of increased control (includes fewer interruptions)**  *Definitely made things easier for the school. Less need for them to manage highs and lows*  *She needs to make less corrections, so has less interruptions in class.* | 5 |
| **Better concentration**  *Less high blood sugar so she can concentrate better. She loses focus when too high.* | 2 |

| **Question 7: How has it changed things during activities? (day to day activity and/or organized sports)** | |
| --- | --- |
| **Response codes and exemplar quotes** (n = 37) | **Frequency counts** |
| **Response did not match the question or no change noticed or no activities** | 23 |
| **Improved control and less interruptions**  *Better control when active*  *It’s made it easier to let her do activities without really much interruption.*  *It has helped him not go low when doing activities.* | 13 |
| **More freedom**  *Gives more freedom to do things* | 1 |
| **Swimming more difficult**  *Not a lot – she moved to this from a tubeless pump, so it makes swimming a bit more difficult.* | 1 |

| **Question 8: How has Control-IQ changed how you feel about diabetes?** | |
| --- | --- |
| **Response codes and exemplar quotes** (n = 37) | **Frequency counts** |
| **More hopeful for future and less worry**  *Gives me hope that he might be independent at some point in the future.*  *I feel she’s very fortunate to have this technology. With her dexcom it’s peace of mind for us. We accept that she has diabetes and are proud on how she has embraced it. This is one more thing that makes her life easier.* | 22 |
| **No change** | 6 |
| **Life is easier**  *I do like how it just automatically controls insulin delivery. Makes things much easier.* | 5 |

| **Question 9: Do you think it has changed anything about how your child/teen feels about diabetes?** | |
| --- | --- |
| **Response codes and exemplar quotes*** (n = 38) | **Frequency counts** |
| **Child feels more positive about diabetes**  *I think it has allowed him to live more normally* | 6 |
| **Child seems less worried**  *I think it makes him worry less.* | 5 |
| **Child has increased independence**  *I think she doesn't feel so restricted now with control IQ.* | 3 |

***Note:** The majority (n=22/38) of responses did not correspond to the question asked or reported no change.

| **Question 10: Has Control-IQ changed how much fun you have together as a family? How you get along with your child/teen? Or the amount of time you spend talking about diabetes? Please tell us more about this.** | |
| --- | --- |
| **Response codes and exemplar quotes** (n = 35) | **Frequency counts** |
| **No change noticed** | 14 |
| **Less talking or nagging about diabetes**  *Yes, I think it has reduced some of the stress in our family. We haven’t needed to nag him as much*  *I feel like I nag her less, mostly about correcting highs.* | 14 |
| **Less time managing diabetes**  *Because I don’t have to be continually making adjustments myself, daily life is much less focused on constantly checking numbers, etc.* | 4 |

| **Question 11: Have you noticed a change in your child/teen? If yes, can you tell us a bit about this?** | |
| --- | --- |
| **Response codes and exemplar quotes** (n = 32) | **Frequency counts** |
| **No change noticed**  *No changed noticed.* | 16 |
| **Increased autonomy**  *[He] has become even more responsible and well educated about management of his diabetes.* | 5 |
| **Better mood**  *Grandparents noticed that he is more happy and calm, and he can enjoy time at their house with no worries like before and no low* | 5 |
